# Effects of Dietary Supplementation with *Lactobacillus reuteri* Postbiotics on Growth Performance, Intestinal Flora Structure and Plasma Metabolome of Weaned Piglets

**DOI:** 10.3390/ani15020204

**Published:** 2025-01-14

**Authors:** Dongfeng Sun, Wenfei Tong, Shaochen Han, Mengjun Wu, Peng Li, Youguo Li, Yunxiang Liang

**Affiliations:** 1 National Key Laboratory of Agricultural Microbiology and College of Life Science and Technology, Huazhong Agricultural University, Wuhan 430070, China; sdf365@126.com; 2 Hubei Key Laboratory of Animal Nutrition and Feed Science, Wuhan Polytechnic University, Wuhan 430023, China; tong1wenfei@163.com (W.T.); 17633859146@163.com (S.H.); wumengjun93@163.com (M.W.); lp1536698031@163.com (P.L.)

**Keywords:** biochemical profiling, microbial diversity, probiotic-derived metabolites, swine

## Abstract

Probiotics and their postbiotics have the potential to improve the health and growth performance of piglets, which has brought them widespread attention in the post-antibiotic era. In the present study, the effects of dietary supplementation of *Lactobacillus reuteri* postbiotics on the growth performance, intestinal flora structure and plasma metabolome of weaned piglets were investigated. A total of 816 healthy male piglets with uniform weight were divided into two treatment groups: piglets in the control (CTR) group were fed with a basic diet, and the ones in the LAC group were fed with the basic diet supplemented with 500 mg/kg *Lactobacillus reuteri* postbiotics. There were six replicates in each group and 68 piglets in each replicate. The animal trial lasted for 30 days. The feces and blood of piglets were collected for investigation, and the growth performance during the trial was counted. Our outcomes show that dietary supplementation with *Lactobacillus reuteri* postbiotics had no effect on the growth performance of piglets; however, it reduced the mortality rate of piglets by 6.37%. The levels of total superoxide dismutase in the serum, propionic acid and butyric acid in the feces were elevated, and the content of malondialdehyde in the serum was decreased with *Lactobacillus reuteri* postbiotics-treated piglets (*p* < 0.05). The fecal flora sequencing results show that the relative abundance of Firmicutes and monoglobus was upregulated, and the relative abundance of Bacteroides was downregulated with *Lactobacillus reuteri* postbiotics-treated piglets (*p* < 0.05). In addition, the levels of propionic acid and butyric acid in the feces were positively correlated with the relative abundance of Firmicutes and negatively correlated with the relative abundance of Bacteroides (*p* < 0.05). The plasma metabolome results show that dietary supplementation with *Lactobacillus reuteri* postbiotics raised the level of coenzyme Q10 in the serum, and the abundance of coenzyme Q10 was positively correlated with the relative abundance of Firmicutes and the level of total superoxide dismutase in the serum. In conclusion, dietary supplementation with *Lactobacillus reuteri* postbiotics contributed to improving the antioxidant function and reducing the mortality of piglets by regulating the structure of intestinal flora and upregulating the content of coenzyme Q10 in serum.

## Simple Summary

Intestinal health is related to the healthy and efficient breeding of piglets, which needs to be focused on in the post-antibiotic era. Microecological agents play an important role in improving the intestinal health of piglets; however, many of the mechanisms have not been characterized. In the present study, we present an updated report of *Lactobacillus reuteri* postbiotics on the growth performance, intestinal flora structure and plasma metabolome of weaned piglets. Our outcomes demonstrate that *Lactobacillus reuteri* postbiotics improve the antioxidant function and reduce the mortality of piglets by regulating the structure of intestinal flora and upregulating the content of coenzyme Q10 in serum. Our findings provide an important theoretical basis for the application of *Lactobacillus reuteri* postbiotics in piglet production and provide new data for the healthy and efficient breeding of piglets.

## 1. Introduction

Previously, antibiotics were allowed to be added to feed due to their beneficial effect on intestinal health in livestock and poultry, as this would help improve growth performance [1]. However, due to the problem of bacterial resistance caused by antibiotics, this could have a negative impact on the health of people. Therefore, the use of antibiotics in feed is no longer permitted. Although livestock products are safer, the intestinal health problems of livestock and poultry are prominent without antibiotics in their diet, and this is particularly evident in young animals [2]. Weaned piglets, for example, are equipped with underdeveloped digestive and immune systems, which make them extremely sensitive to the external environment, such as abnormal environmental changes, pathogen infection, transportation and other stress [3]. The development of safe and effective alternatives to antibiotics in feed and the enhancement of piglets’ ability to respond to environmental stress is one of the key topics in the current research on livestock.

It is well known that antibiotics in feed can regulate the structure of intestinal flora, such as inhibiting the proliferation of harmful bacteria and elevating the abundance of beneficial bacteria, thus improving the intestinal physiological function of livestock [4], while developing antibiotic substitutes helps to regulate the intestinal flora structure and improve the intestinal function of livestock in the post-antibiotic era [5]. Hence, we should pay more attention to the intestinal flora of livestock reared on feed with and without the use of antibiotics. Studies have demonstrated that probiotics and their postbiotics are able to improve the growth performance and health of piglets by improving the structure of intestinal flora and regulating the immune and antioxidant functions of piglets [5,6]. Among them, *Lactobacillus reuteri* is a lactobacillus that has been reported to colonize the intestines of almost all vertebrates and mammals [7]. Therefore, *Lactobacillus reuteri* is safe for animals and humans, which is the main reason why it is widely studied and used in medicine and food. A study demonstrated that *Lactobacillus reuteri* could metabolize and produce bioactive substances, such as short-chain fatty acids and indole-3-acetaldehyde, which contribute to improving intestinal health and growth performance [8,9]. However, the application of postbiotics developed based on the metabolites of *Lactobacillus reuteri* and its bacterial composition in pig production is rarely reported.

We believed it would be interesting to study the effects of *Lactobacillus reuteri* postbiotics on the growth performance and intestinal flora structure of piglets. In the present study, *Lactobacillus reuteri* postbiotics were used to conduct experiments in large populations of piglets and harvest samples based on non-invasive sampling methods to investigate the effects of dietary supplementation of *Lactobacillus reuteri* postbiotics on the growth performance, intestinal flora structure and plasma metabolome of weaned piglets. The purpose of this study was to provide a theoretical basis for the application of *Lactobacillus reuteri* postbiotics in the healthy and efficient breeding of piglets.

## 2. Materials and Methods

### 2.1. Experiment Design and Animal Management

A single-factor experimental design was implemented to perform a 30-day animal trial in the breeding farm of New Hope in Zhenyuan, Guizhou, China. The 30-day use period was based on our experience with over 2000 broiler chickens and our results with over 20 weaned piglets (unpublished data). The 30-day animal trial period was chosen for the following reasons: our preliminary experimental results demonstrated that 30 days of *Lactobacillus reuteri* postbiotics supplementation was sufficient to improve the health of piglets. In addition, considering that the cost of *Lactobacillus reuteri* postbiotics per ton of feed addition might be higher than CNY 30, long-term use would increase the breeding cost, and the experimental protocol and management of the pigs were approved by the Animal Care and Use Committee of New Hope Liuhe Co., Ltd., (Chengdu, China). The code for ethical inspection was IAS 2023-32. A total of 816 healthy male weaned piglets (those pigs with the same genetic background of Durc× Landrace× Yorkshire were weaned at 21 days of age after birth and started to be kept in corrals on a diet of feed for a 3-day acclimation period. With uniform body weight (7.06 ± 0.73 kg), they were randomly divided into the control group (CTR) and *Lactobacillus reuteri* postbiotics group (LAC). There were 6 replicates in each treatment and 68 piglets in each replicate. Piglets in the control group were fed a basic diet, and the diet formula was formulated according to the NRC 2012 nutritional requirements standard for piglets, and the formula and nutritional composition are shown in Table 1. Piglets in the LAC group were fed with the basic diet supplemented with 500 mg/kg *Lactobacillus reuteri* postbiotics purchased from Hubei Lan Good Microbial Technology Co. Ltd., Yichang, Hubei, China. The control group diet was correspondingly supplemented with an equal volume of a *Lactobacillus reuteri* postbiotics carrier (wheat bran and zeolite powder were mixed according to a 1:1 mass ratio). During the animal trial, piglets in each group were fed the same batch of feed without any change, all piglets were free to feed and water, and the routine immunization procedures were carried out according to the requirements of the farm. Briefly, all piglets were raised in a room, with each of the 68 piglets housed individually in a concrete enclosure without bedding (20 × 40 m^2^), and the room temperature was maintained at 25 ± 2 °C, and a 24-hour lighting schedule was implemented. The number of piglet deaths during the animal trial was recorded, and all piglets were weighed at the end of the trial, and the feed consumption was measured. At the same time, two pigs from each replicate group were selected to collect blood from the anterior vena cava for the detection of antioxidant-related indexes and metabolome. Fresh feces were collected at the end of the animal trial to investigate the intestinal flora structure and levels of short-chain fatty acids.

### 2.2. Growth Performance

The average daily gain (ADG) is expressed as the difference between the weight at the end and the weight at the start divided by the number of days in the trial. The average daily feed intake (ADFI) is characterized by the amount of feed consumed during the animal trial period divided by the number of days tested. The ratio of ADFI to ADG indicates the feed conversion efficiency (FCR), and the ratio of the number of piglets that died during the trial to the number of piglets at the start of the trial is indicative of piglet mortality.

### 2.3. Blood Biochemical Indexes and Antioxidant Related Parameters

Blood samples were collected from the anterior vena cava into either heparinized, and then they was centrifuged for 10 min at 3500 r/min and 4 °C to harvest the plasma. A Hitachi 7060 Automatic Biochemical Analyzer (Hitachi, Japan) was used to measure the blood biochemical indexes shown in Table 2. The contents of the antioxidant enzymes and peroxide products, for instance, superoxide dismutase (SOD), malondialdehyde (MDA), myeloperoxidase (MPO), and glutathione peroxidase (GSH-px), were investigated according to the steps of the kit purchased from Nanjing Jiancheng Bioengineering Institute, Nanjing, China.

### 2.4. Levels of Short-Chain Fatty Acids in Feces

Fresh feces were collected and placed in a sterile EP tube and frozen with liquid nitrogen, then transferred into a −80 °C refrigerator for storage. The contents of short-chain fatty acids in feces were determined according to the method described by Li et al. [10]. Briefly, 0.5 g of feces was weighed and placed in a clean EP tube containing 1.5 mL of ultra-pure water, and then it was mixed well and left to rest for 30 min. Next, 1 mL of supernatant was collected after centrifugation at 4 °C and 15,000 r/min for 20 min. The supernatant was transferred into a new 2 mL EP tube containing 0.2 mL 25% metaphosphates solution, which was mixed well and centrifuged again under the same conditions as above to collect the supernatant, and then the supernatant was passed through a 0.22-micron filter membrane, and the filtrate was taken to be measured. A gas chromatography analyzer (Agilent GC-MS 7890B) was used to detect the levels of acetic acid, propionic acid and butyric acid. The chromatographic column was an Agilent DB-FFAP, the chromatographic column parameter was 30 m × 0.25 mm × 0.25 μm, and the carrier gas was helium with purity greater than 99.99%. In the process of gas phase implementation, a sample size of 1 μL was required for treatment at 50 °C for 1 min, followed by heating at 10 °C/min to 200 °C. The inlet temperature was 250 °C, and the flow rate was 1.0 mL/min. EI was used as the ionization mode, the electron energy was −70 eV, and the shunt ratio was controlled at 2:1. Additionally, the temperature of the ion source and the transmission line were maintained at 280 °C, 0.954 kV was a must as the electron multiplier voltage, and 150 °C was needed in the four-stage rod temperature. These test conditions guaranteed a scanning range of 33–200 *m*/*z*. Finally, data were analyzed with the agilent mass hunter.

### 2.5. 16S Sequencing and Analysis

The fresh feces were harvested at the end of the animal trial and frozen with liquid nitrogen, then transferred into a −80 °C refrigerator for storage. The 16S sequencing and analysis were implemented according to the method described by Li et al. [11]. Briefly, the bacterial DNA was extracted, its concentration and purity were measured, and the V3–V4 region was amplified using a universal primer and named 515 F and 806 R of the 16S rDNA gene. After the mixing and purification of PCR products were performed, the sequencing library was formed by terminal repair, the addition of the A-tail, and the addition of sequencing joints. An Illumina HiSeq2500 PE250 platform (Illumina, San Diego, CA, USA) was used to perform sequencing at Novogene Bioinformatics Technology Co., Ltd., Beijing, China. The raw data were spliced and filtered to obtain clean data, and then the final ASVs were obtained through DADA2 based on the clean data, and species annotation was made based on the ASVs, and the relative abundance, Alpha diversity calculation, Venn diagram and petal diagram were analyzed. LEFse software (Version 1.0) was used to analyze the dominant flora between groups, and R (Version 2.15.3) was used to perform a T-test analysis between the two groups to obtain differential flora. Finally, PICRUST2 functional prediction analysis was used to implement the functional prediction of differences between groups at Level 3 to obtain valuable information for the study. The original data of 16S sequencing have been uploaded to the NCBI database with the login number PRJNA1195387.

### 2.6. Plasma Metabolome

Blood samples were collected from the anterior vena cava into either heparinized, and then they were centrifuged for 10 min at 3500 r/min and 4 °C to harvest the plasma. A liquid chromatography–tandem mass spectrometry (LC-MS/MS) (ultra-high liquid chromatograph, LC-30A, Shimadzu, Japan, and mass spectrometer: TripleTOF 6600+, SCIEX, Foster City, CA, USA) platform was used to investigate the plasma metabolome. Specifically, 50 μL plasma was added into a clean EP tube containing 300 μL of 20% acetonitrile–methanol internal standard extraction solution and mixed for 3 min and centrifuged at 4 °C and 12,000 r/min for 10 min to obtain 200 μL of the supernatant. The supernatant was placed in a −20 °C refrigerator for 30 min, and then 180 μL of the supernatant was obtained by centrifugation at 4 °C and 12,000 r/min for 3 min and analyzed with the supernatant. Under chromatographic conditions, the Water ACQUITR Premier HSS T3 column (1.8 μm and 2.1 mm × 100 mm) was used, and mobile phase A was a 0.1% formic acid aqueous solution and mobile phase B was a 0.1% formic acid acetonitrile solution. The column temperature was 40 °C, the flow rate was 0.4 mL/min, and the sample size was 4 μL. The mass spectrum conditions of the AB TripleTOF 6600 are shown in Table 3. The samples were extracted and tested by Wuhan Meiwei Metabolic Biotechnology Co., Ltd. Wuhan, China. The original data format of the mass spectrometry machine needed to be converted to mzXML, and XCMS sequencing was used to perform peak extraction, alignment, filtration and retention time correction and then identify metabolites based on the mtDNA method with the Meiwei metabolic database and integrated public database. Finally, a list of all differential metabolites was obtained based on the criterion that the CV value of QC (quality control) samples was less than 0.3. R (Version 2.15.3) was used for PCA and OPLS-DA analysis, and Volcano maps with differential metabolite bar plots were drawn based on a VIP greater than 1. The top 50 metabolites with the largest VIP (variable importance in projection) values were used to map the chord, and the typical differential metabolites of interest were used for independent sample T-test analysis.

### 2.7. Statistical Analysis

All data were analyzed by one-way ANOVA procedure in the SPSS 23.0 software (SPSS Inc. Chicago, IL, USA) and expressed as mean ± SD, and the independent sample T-test was used to analyze the differences between groups. In addition, Pearson correlation analysis in the SPSS 23.0 software (SPSS Inc., Chicago, IL, USA) was used to investigate correlations between different flora and observed indicators, as well as between different metabolites and different bacteria or observed indicators. A value of *p* < 0.05 was taken to indicate statistical significance. If necessary, the first author can be contacted by email regarding the original data of the full text.

## 3. Results

### 3.1. Effects of Lactobacillus reuteri Postbiotics on Growth Performance and Blood Biochemical Indices of Weaned Piglets

**Figure 1:**
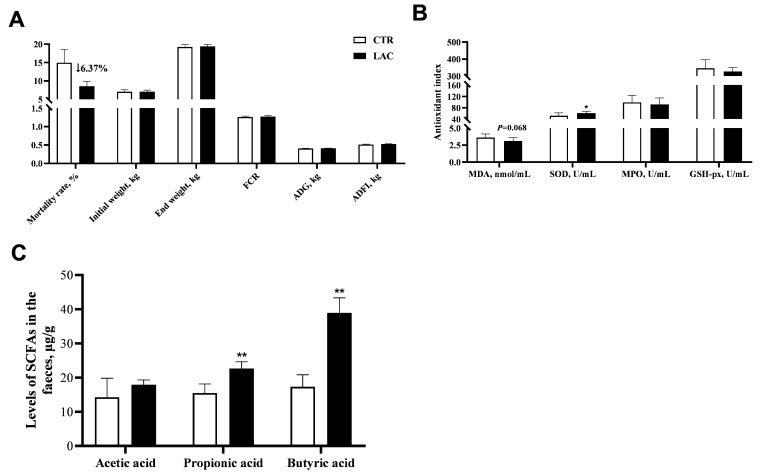
Effects of *Lactobacillus reuteri* postbiotics on growth performance, plasma antioxidant parameters and fecal short-chain fatty acid content of piglets. Among them, results of growth performance are arranged in (**A**), the antioxidant parameters in plasma are shown in (**B**), and levels of short-chain fatty acids are arranged in (**C**). FCR represents feed conversion efficiency, ADFI represents average daily feed intake, ADG represents average daily gain, MDA represents malondialdehyde, SOD represents superoxide dismutase, MPO represents myeloperoxidase, and GSH-px represents glutathione peroxidase. * 0.01 < *p* < 0.05, and ** 0.001 < *p* < 0.01.

Compared with the control group, dietary supplementation with *Lactobacillus reuteri* postbiotics had no significant effect on the growth performance of piglets. However, it is worth mentioning that although we did not observe a statistical difference, *Lactobacillus reuteri* postbiotics treatment reduced the mortality of weaned piglets by 6.37% (Figure 1A). In addition, dietary supplementation with *Lactobacillus reuteri* postbiotics did not cause abnormal changes in the blood biochemical indices (Table 2).

### 3.2. Effects of Lactobacillus reuteri Postbiotics on Antioxidant-Related Parameters in Plasma and Levels of Short-Chain Fatty Acids in Feces

Dietary supplementation with *Lactobacillus reuteri* postbiotics tended to decrease the level of MDA (*p* = 0.068) and significantly raised the content of SOD (*p* < 0.05). Additionally, the levels of propionic acid and butyric acid in the feces were elevated in the diet treated with *Lactobacillus reuteri* postbiotics (*p* < 0.05) (Figure 1B,C).

### 3.3. Effects of Lactobacillus reuteri Postbiotics on Intestinal Flora Structure of Feces

**Figure 2:**
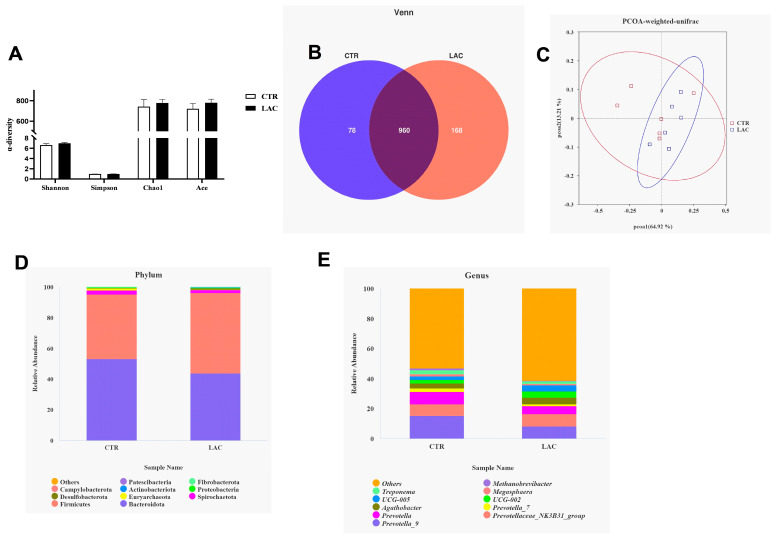
Effects of *Lactobacillus reuteri* postbiotics on fecal flora structure of piglets. Among them, results of α-diversity are arranged in (**A**), the Venn and PCOA results are shown in (**B**) and (**C**), respectively. (**D**) and (**E**) show the relative abundance of the top ten bacteria at phyla level and genus level, respectively.

**Figure 3:**
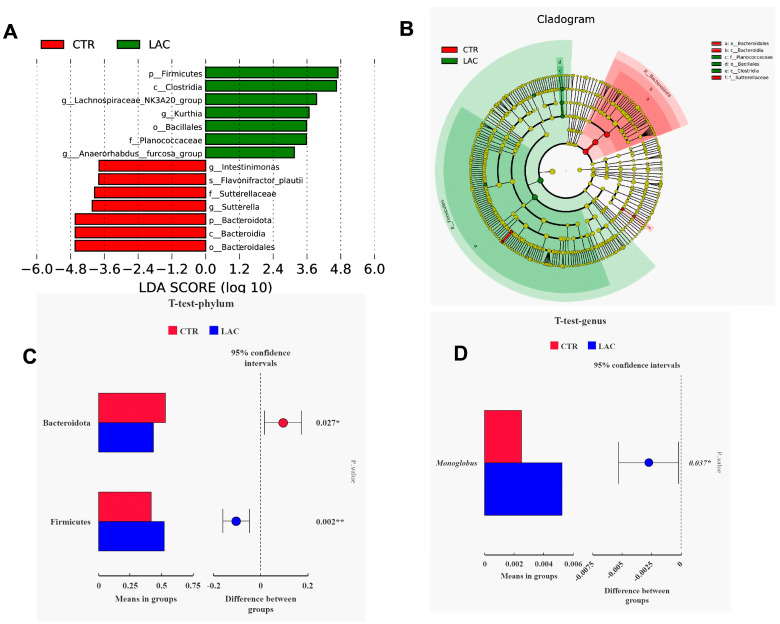
Results of flora structure difference between CTR and LAC group based on LEFse and T-test analyses. Among them, results based on LEFse analysis are shown in (**A**,**B**), and outcomes based on T-test analysis are arranged in (**C**,**D**). * 0.01 < *p* < 0.05, and ** 0.001 < *p* < 0.01.

Dietary supplementation with *Lactobacillus reuteri* postbiotics had no effect on the α-diversity of intestinal flora, and the number of OTUs unique to the LAC group was 168, and the number of OTUs shared with the control group was 960. Additionally, dietary supplementation with *Lactobacillus reuteri* postbiotics upregulated the relative abundance of Firmicutes and downregulated the relative abundance of Bacteroidetes (Figure 2). The outcomes based on the LEFse analysis show that Firmicutes and *Lachnospiraceae-NK3A20* were the dominant bacteria in the LAC group, and Bacteroidetes was the dominant bacteria in the CTR group (Figure 3A,B). The T-test analysis further confirmed that *Lactobacillus reuteri* postbiotics contributed to upregulating the relative abundance of Firmicutes and downregulating the relative abundance of Bacteroidetes (Figure 3C,D).

**Figure 4:**
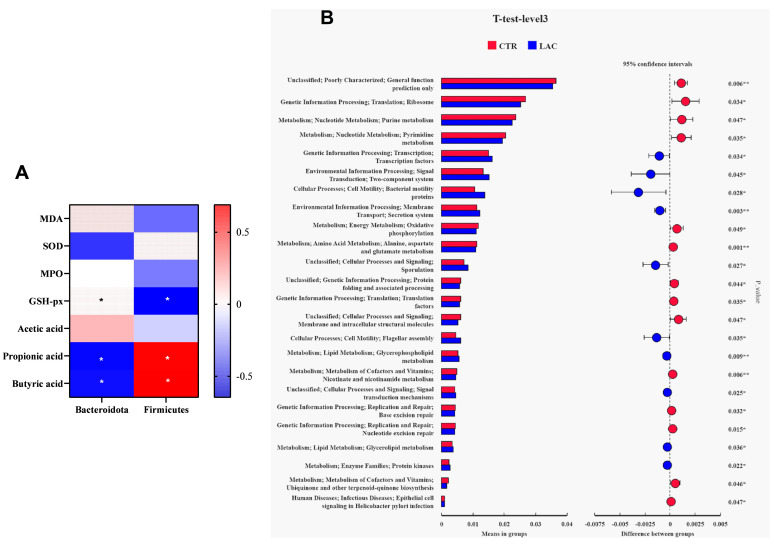
Results of correlation analysis and functional prediction. Among them, results of correlation analysis are arranged in (**A**), and (**B**) shows outcomes of functional prediction. MDA represents malondialdehyde, SOD represents superoxide dismutase, MPO represents myeloperoxidase, GSH-px represents glutathione peroxidase. * 0.01 < *p* < 0.05, and ** 0.001 < *p* < 0.01.

The outcomes of the correlation analysis show that the contents of propionic acid and butyric acid in the feces were positively correlated with the relative abundance of Firmicutes and negatively correlated with the relative abundance of Bacteroidetes (*p* < 0.05). The functional prediction results demonstrate that *Lactobacillus reuteri* postbiotics enhanced bacterial signal transduction, bacteria motility proteins, secretory system, lipid metabolism and other functions (Figure 4).

### 3.4. Effects of Lactobacillus reuteri Postbiotics on Plasma Metabolome

**Figure 5:**
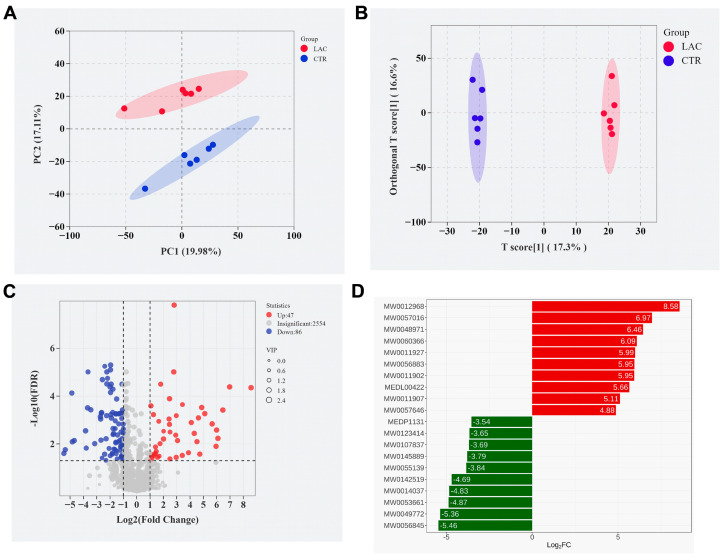
Effects of Lactobacillus reuteri postbiotics on serum metabolome. Among them, results of PCA and OPLS-DA analyses are arranged in (**A**,**B**), the volcanic maps are shown in (**C**), and typical differential metabolite bars are arranged in (**D**). 1-hexadecanoyl-2-(9Z,12Z-octadecadienoyl)-sn-glycero-3-phosphocholine = MW0012968, 1-oleoyl-2-palmitoyl-sn-glycero-3-phosphocholine = MW0057016, coenzyme Q10 = MW0048971, PE-NMe2 (18:1(9Z)/18:1(9Z)) = MW0060366, and 1,2-distearoyl-sn-glycero-3-phosphocholine = MW0011927. 5-Hydroxy-6-methoxy-3-methyl-2-octaprenyl-1,4-benzoquinone = MW0142519, 3-hexanoyl-NBD cholesterol= MW0014037, glucocerebrosides = MW0053661, and 1-palmitoyl-3-adrenoyl-sn-glycerol = MW0049772, PC (14:1(9Z)/P-18:1(11Z)) = MW0056845. Red represents upregulation, and green represents downregulation.

The metabolite composition in the plasma of the LAC and CTR piglets was inconsistent. Specifically, dietary supplementation with *Lactobacillus reuteri* postbiotics raised 47 metabolites and decreased 86 metabolites. The typical upregulated metabolites were 1-hexadecanoyl-2-(9Z,12Z-octadecadienoyl)-sn-glycero-3-phosphocholine (MW0012968), 1-oleoyl-2-palmitoyl-sn-glycero-3-phosphocholine (MW0057016), coenzyme Q10 (MW0048971), PE-NMe2 (18:1(9Z)/18:1(9Z)) (MW0060366) and 1,2-distearoyl-sn-glycero-3-phosphocholine (MW0011927). The typical downregulated metabolites were 5-Hydroxy-6-methoxy-3-methyl-2-octaprenyl-1,4-benzoquinone (MW0142519), 3-hexanoyl-NBD cholesterol (MW0014037), glucocerebrosides (MW0053661), 1-palmitoyl-3-adrenoyl-sn-glycerol (MW0049772) and PC(14:1(9Z)/P-18:1(11Z)) (MW0056845) (Figure 5).

**Figure 6:**
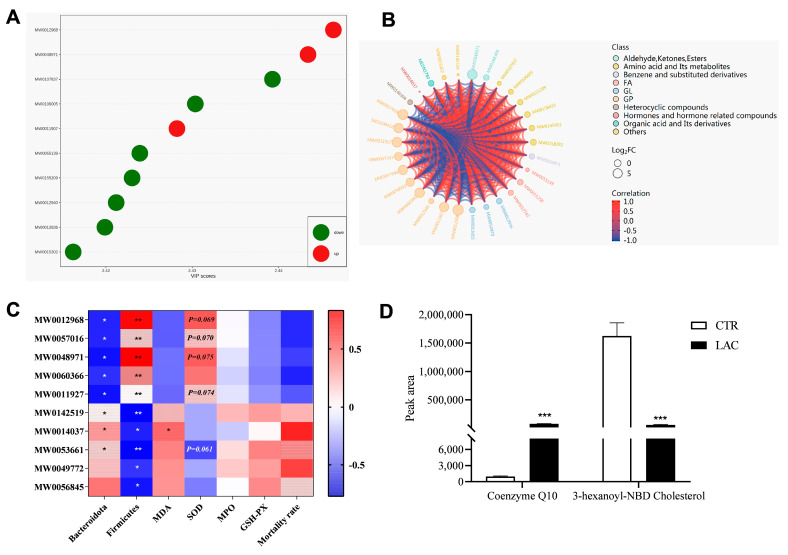
Analysis results of typical differential metabolites and their association with the indicators detected in the present study. Among them, VIP value diagram of differential metabolites and correlation chord diagram of metabolites are arranged in (**A**) and (**B**), respectively. Results of typical differential metabolites and their association with the indicators detected in the present study are shown in (**C**), and the results of peak area regarding coenzyme Q10 and 3-hexanoyl-NBD cholesterol are arranged in (**D**). MDA represents malondialdehyde, SOD represents superoxide dismutase, MPO represents myeloperoxidase, and GSH-px represents glutathione peroxidase. * 0.01 < *p* < 0.05, ** 0.001 < *p* < 0.01, and *** *p* < 0.001.

The differences in metabolites were mainly concentrated in glycerophospholipids and sphingolipids, as well as ketones and hormones. The outcomes of the correlation analysis show these upregulated metabolite levels were positively correlated with the relative abundance of Firmicutes and the content of SOD in plasma and negatively correlated with the relative abundance of Bacteroidetes. This was the opposite in the metabolites that were downregulated (*p* < 0.05). The two different metabolites we focused on were coenzyme Q10 and 3-hexanoyl-NBD cholesterol, and dietary supplementation with *Lactobacillus reuteri* postbiotics elevated the level of coenzyme Q10 and decreased the level of 3-hexanoyl-NBD cholesterol in the plasma of the piglets (*p* < 0.05) (Figure 6).

## 4. Discussion

The weaning stage needs to be paid more attention in the healthy and efficient breeding process of pigs since the immune system and digestive system of the gastrointestinal tract of weaned piglets are not fully developed, which makes the piglets less resistant to exogenous stressors [3]. In the breeding process of piglets, pathogen infection, abnormal environmental stimulation, anti-nutrient factors in feed and other exogenous stressors enhance the negative effects of weaning stress on piglets [12,13]. These are the key reasons for the poor growth performance and high mortality of piglets at the weaning stage. As we observed in the present study, the mortality rate of piglets was close to 15%. Hence, it is a top priority for farmers or pig enterprises to reduce mortality and improve growth performance. Studies have demonstrated that dietary supplementation with *Lactobacillus reuteri* improved the intestinal flora structure, reduced the diarrhea rate, and then improved the growth performance of piglets [14,15,16,17]. Paradoxically, the beneficial effects of *Lactobacillus reuteri* were not always stable and might be closely related to the source of the strain and its ability to colonize the animal. Postbiotics, which are non-living active ingredients that contribute to improving the unstable efficacy of probiotics, have been extensively studied in recent years [16,17]. In the present study, although dietary supplementation with *Lactobacillus reuteri* postbiotics had no effect on the growth performance of weaned piglets, it was interesting to note that mortality was reduced by 6.37%. We suggest this will likely be highly welcomed by pig producers. In addition, the standard deviation of piglet mortality values in the LAC treatment group was smaller. This indicates that the *Lactobacillus reuteri* postbiotics intervention results in piglets showing better uniformity, which is also of interest to farmers. Subsequent experimental investigations were carried out to explain the reason for the beneficial effects of *Lactobacillus reuteri* postbiotics on the piglets.

Levels of biochemical markers in the blood could characterize the health of the body, such as elevated levels of glutamic oxalacetic transaminase and glutamic pyruvic transaminase, which indicate that the liver could be damaged [18]. Any exogenous factors might cause abnormal changes in the levels of biochemical indexes in blood, which would be a burden on the body. This justifies why we should not blindly take supplements [19]. In the present study, dietary supplementation with *Lactobacillus reuteri* postbiotics had no effect on the levels of biochemical indexes in the blood. This indicates that *Lactobacillus reuteri* postbiotics are clearly safe for piglets. With stimulated stressors, oxygen free radicals and peroxide products were expressed in large quantities in the piglets, which caused an imbalance between the oxidation and antioxidant systems. In order to maintain the balance of this system, the antioxidant system was activated, and then some antioxidant enzymes, such as SOD and GSH-px, were expressed to remove peroxide products, for instance, MDA or oxygen free radicals [20]. In the present study, dietary supplementation with *Lactobacillus reuteri* postbiotics raised the level of SOD and decreased the content of MDA. These outcomes illuminate that *Lactobacillus reuteri* postbiotics have the potential to relieve the oxidative stress of weaned piglets, and this might be one of the reasons why *Lactobacillus reuteri* postbiotics reduced the mortality of piglets in our present study.

A study demonstrated that *Lactobacillus reuteri* could metabolize to produce short-chain fatty acids, which helped to improve the antioxidant function and health status of piglets [21]. In this study, we also found that *Lactobacillus reuteri* postbiotics upregulated the contents of propionic acid and butyric acid in the feces of piglets. Although we did not observe the relevant indicators of intestinal function, the upregulation of short-chain fatty acids caused by *Lactobacillus reuteri* postbiotics intervention might be helpful to the intestinal health of piglets, which might be more evidence that dietary supplementation with *Lactobacillus reuteri* postbiotics contributed to reducing the mortality of piglets. We were willing to attribute that *Lactobacillus reuteri* postbiotics raised the levels of fecal short-chain fatty acids, improving the intestinal flora structure. The outcomes of the intestinal flora structure in the present study also confirmed our hypothesis. As we observed, dietary supplementation with *Lactobacillus reuteri* postbiotics upregulated the relative abundance of Firmicutes and downregulated the relative abundance of Bacteroidetes, which also showed a significant correlation with the changing trend of short-chain fatty acids. This study demonstrates that most of the bacteria in Firmicutes have the ability to metabolize and produce short-chain fatty acids, and the increased ratio of Firmicutes to Bacteroides might be more beneficial to the intestinal health of piglets [22]. Our outcomes suggest the potential of *Lactobacillus reuteri* postbiotics in improving intestinal health in piglets and that further research is worth pursuing in this area.

A study demonstrated that *Lachnospiraceae-NK3A20* might contribute to the metabolism of amino acids and glycerophospholipids, enhancing the antioxidant function of the body [23]. *Monoglubos*, a bacterium that degrades pectin to produce short-chain fatty acids, might be useful in maintaining immune homeostasis [24]. In the present study, *Lactobacillus reuteri* postbiotics upregulated the relative abundance of *Lachnospiraceae-NK3A20* and *Monoglubos* and enhanced the amino acid and lipid metabolism functions. In the present study, the correlation analysis between the outcomes of bacteria sequencing and serum metabolites shows that the changes in Firmicutes and Bacteroidetes induced by *Lactobacillus reuteri* postbiotics were significantly correlated with major differential metabolites. The correlation analysis further shows that these different metabolites were significantly correlated with the level of SOD in the plasma. These results are logically consistent, and they all point to the hypothesis that *Lactobacillus reuteri* postbiotics might improve the health of the body by regulating the structure of intestinal flora.

In addition to playing a key role in mitochondrial oxidative phosphorylation, coenzyme Q10 also acts as a fat-soluble antioxidant, plays an important role in fatty acid, pyrimidine and lysosome metabolism, and directly mediates the expression of many genes, including those associated with inflammation [25]. In the present study, dietary supplementation with *Lactobacillus reuteri* postbiotics raised the level of coenzyme Q10 in the plasma, and its abundance was positively correlated with the level of SOD in the plasma. This illuminates that the beneficial effect of *Lactobacillus reuteri* postbiotics on antioxidant function might be related to its upregulation of coenzyme Q10. Based on all the outcomes in the present study, we conclude that *Lactobacillus reuteri* postbiotics might improve the survival rate of piglets by reshaping the structure of intestinal flora and plasma metabolome. Although a detailed investigation of the regulatory mechanism of *Lactobacillus reuteri* postbiotics on intestinal health was beyond the scope of this work, we acknowledge that *Lactobacillus reuteri* postbiotics have great application potential in healthy piglet breeding.

## 5. Conclusions

Dietary supplementation with *Lactobacillus reuteri* postbiotics improved the survival rate of piglets by regulating the structure of intestinal flora and reshaping plasma metabolome, especially upregulating the content of coenzyme Q10.

## Figures and Tables

**Table 1:** Feed formulation and nutritional value on dry matter basis.

Feed Formulation			Nutrient Level ^3^		
Ingredients	CTR	LAC	Item	CTR	LAC
Corn	31.10	31.10	NE, kcal	2570	2570
Soybean meal	11.00	11.00	DE, kcal	3509	3509
Extruded soybean	10.00	10.00	CP, %	17.70	17.70
Wheat germ	4.00	4.00	EE, %	5.79	5.79
Extruded corn	20.00	20.00	CF, %	2.82	2.82
Whey powder	7.50	7.50	NDF, %	8.09	8.09
Fermented soybean meal	5.00	5.00	Ca, %	0.62	0.62
White sugar	2.00	2.00	total P, %	0.60	0.60
Wheat bran	1.70	1.70	Ash, %	4.77	4.77
Coconut oil powder	1.25	1.25			
Soybean oil	1.00	1.00			
Ca(H_2_PO_4_)_2_	0.89	0.89			
L-lysine	0.66	0.66			
Calcium formate	0.50	0.50			
Acidifying agent	0.50	0.50			
Stone powder	0.40	0.40			
L-valine	0.36	0.36			
L-threonine	0.31	0.31			
DL-methionine	0.31	0.31			
Montmorillonite	0.45	0.45			
L-tryptophan	0.27	0.27			
Trace element premix ^1^	0.25	0.25			
Zinc oxide	0.18	0.18			
NaCl, 98.5%	0.18	0.18			
Choline chloride, 60%	0.08	0.08			
Vitamin premix ^2^	0.05	0.05			
Phytase, 20,000 IU	0.03	0.03			
Sandoquine	0.02	0.02			
Sweetening agent	0.01	0.01			
*Lactobacillus reuteri* postbiotics	0.00	0.05			

^1^ In the trace mineral elements, the content of copper was 40,000 mg/kg, the content of iron was 75,000 mg/kg, the content of zinc was 30,000 mg/kg, and the content of manganese was 35,000 mg/kg. ^2^ The main elements in vitamin premix were as follows: VA: 20 million IU/kg, VD_3_: 10 million IU/kg, VE: 100,000 mg/kg, VK_3_: 10,000 mg/kg, VB_1_: 5000 mg/kg, VB_2_: 12,000 mg/kg, VB_6_: 4000 mg/kg and Niacinamide: 60,000 mg/kg. ^3^ Calculation level.

**Table 2:** Results of serum biochemical index.

Item	CTR	LAC	*p*-Value
TB, μmol/L	0.30 ± 0.13	0.31 ± 0.12	0.979
TP, g/L	61.13 ± 2.90	57.91 ± 1.00	0.317
ALB, g/L	34.57 ± 1.74	32.34 ± 0.88	0.285
GLB, g/L	26.56 ± 1.49	25.57 ± 1.05	0.595
AST, U/L	73.20 ± 17.14	59.51 ± 3.09	0.451
ALT, U/L	44.70 ± 3.53	43.44 ± 3.58	0.806
ALP, U/L	320.50 ± 18.21	315.70 ± 14.14	0.837
TC, mmol/L	2.38 ± 0.09	2.31 ± 0.10	0.637
TG, mmol/L	0.49 ± 0.03	0.43 ± 0.05	0.263
GLU, mmol/L	5.51 ± 0.25	5.42 ± 0.21	0.796
CA, mmol/L	3.20 ± 0.13	3.06 ± 0.07	0.368
P, mmol/L	2.99 ± 0.26	3.24 ± 0.13	0.420
CREA, μmol/L	72.22 ± 4.14	77.73 ± 4.91	0.400
HDL, mmol/L	0.87 ± 0.04	0.85 ± 0.05	0.813
LDL, mmol/L	1.19 ± 0.05	1.11 ± 0.06	0.274
GGT, U/L	53.13 ± 3.65	59.23 ± 3.02	0.213
CK, U/L	2261.18 ± 230.19	2049.52 ± 238.95	0.532
LDH, mmol/L	1116.80 ± 139.45	991.04 ± 85.45	0.443

TB = total bilirubin, TP = total protein, ALB = albumin, GLB = globulin, AST = glutamic oxalacetic transaminase, ALT = glutamic–pyruvic transaminase, ALP = alkaline phosphatase, TC = total cholesterol, TG = triglyceride, GLU = glucose, CA = calcium, P = phosphorus, CREA = creatinine, HDL = high-density lipoprotein, LDL = low-density lipoprotein, GGT = glutamyltranspeptidase, CK = creatine kinase, and LDH = lactate dehydrogenase.

**Table 3:** The mass spectrum conditions of AB TripleTOF 6600.

Item	ESI+	ESI−
Duration, min	10	10
Ion Spray Voltage, V	5000	−4000
Temperature, °C	550	450
Ion Source Gas 1, psi	50	50
Ion Source Gas 2, psi	60	60
Curtain Gas, psi	35	35
Declustering Potential, V	60	−60
MS1 Collision Energy, V	10	−10
MS2 Collision Energy, V	30	−30
Collision Energy Spread, V	15	15
MS1 TOF Masses, Da	50–1000	50–1000
MS2 TOF Masses, Da	25–1000	25–1000
MS1 Accumulation Time, s	0.2	0.2
MS2 Accumulation Time, s	0.04	0.04
Candidate Ions	18	18

## Data Availability

If necessary, the first author can be contacted by email regarding the original data of the full text.
